# Reductions in Postprandial Plasma Allantoin Concentrations With Increasing Doses of Polyphenol Rich Curry Intake – A Randomized Crossover Trial

**DOI:** 10.3389/fphys.2018.01899

**Published:** 2019-01-09

**Authors:** Sumanto Haldar, Leroy Sivappiragasam Pakkiri, Joseph Lim, Siok Ching Chia, Shalini Ponnalagu, Chester Lee Drum, Christiani Jeyakumar Henry

**Affiliations:** ^1^Clinical Nutrition Research Centre, Singapore Institute for Clinical Sciences, Agency for Science, Technology and Research, Singapore, Singapore; ^2^Cardiovascular Research Institute, National University Health System, Singapore, Singapore; ^3^Department of Medicine, Yong Loo Lin School of Medicine, National University of Singapore, Singapore, Singapore; ^4^Department of Biochemistry, National University of Singapore, Singapore, Singapore

**Keywords:** allantoin, F2-isoprostanes, peripheral arterial tonometry, spices, polyphenols

## Abstract

While dietary or supplementary antioxidants are thought to inhibit or delay oxidation of biological molecules, their utility *in vivo* has been marred by equivocal evidence. Consumption of polyphenol rich foods has been thought to alleviate postprandial oxidative stress and/or improve endothelial function. Although, previous studies suggested the utility of allantoin as a biomarker of oxidative stress, controlled dose response studies with dietary antioxidants to test this in humans have been limited. We therefore investigated the effects of 2 doses of polyphenol rich curry consumption on postprandial plasma concentrations of allantoin, allantoin to uric acid ratio, F2-isoprostanes using liquid chromatography-tandem mass spectrometry (LCMS-MS) and measured endothelial function using peripheral arterial tonometry (endoPAT). In a randomized controlled crossover trial in 17 non-smoking, healthy, Chinese men, aged 23.7 ± 2.4 years and BMI 23.1 ± 2.3 kg/m^2^, the volunteers consumed 3 test meals in a random order, consisting of either non-curry Dose 0 Control (D0C, 0 g spices), or Dose 1 Curry (D1C, 6 g spices) or Dose 2 Curry (D2C, 12 g spices), after overnight fast. There were significant reductions in postprandial allantoin concentrations (*p* < 0.001) and allantoin to uric acid ratio (*p* < 0.001) at 2 h and 3 h following test meal consumption, indicating improvements in postprandial redox balance with increasing curry doses, although there were no differences between treatments on F2-isoprostane concentrations or on RHI (measured at 2 h only). Allantoin may have a utility as a biomarker of redox balance, in an acute setting. The study was registered at www.clinicaltrials.gov (Identifier No. NCT02599272).

## Introduction

A number of recent studies have shown that the consumption of polyphenol-rich foods can simultaneously improve markers of postprandial oxidative stress (Umeno et al., 2016), markers of inflammatory response (Cerletti et al., 2015) as well as vascular function (Storniolo et al., 2014). Such foods include chocolate (Mellor et al., 2012), green tea (Suzuki-Sugihara et al., 2016), coffee (Kajikawa et al., 2018), olive oil (Weinbrenner et al., 2004), and even mixed polyphenol rich foods (Annuzzi et al., 2014). On the contrary, other studies found no significant effect of polyphenol intake and markers of oxidative stress (Wang et al., 2000; Widlansky et al., 2005; Ziegler et al., 2005). The heterogeneity in the study findings are partly due to the types and doses of polyphenols (or other antioxidants) used in the various studies, as well as differences in the study populations investigated upon. Moreover, markers of oxidative stress/redox balance used in the different studies have also been rather variable and given the large inter-individual variability in polyphenol metabolism and the small numbers of participants involved, some of these studies could well have been underpowered. All of these have led to several controversies regarding the roles and utilities of dietary (exogenous) antioxidants, defined as “substances in foods that significantly decreases the adverse effects of reactive oxygen species, reactive nitrogen species, or both on normal physiological function in humans” (Institute of Medicine Standing Committee on the Scientific Evaluation of Dietary Reference Institut, 1998), including polyphenols, in improving redox balance in healthy participants (Touyz, 2004). Moreover, recent reviews and commentaries in fact have argued against the direct antioxidant role of dietary polyphenols *in vivo*, suggesting instead, alternative mechanisms through which dietary polyphenol intake appears to benefit metabolic functions which include modulation of gene expression, cell signaling, inflammation, mitochondrial function, insulin resistance, glucose, and lipid metabolism (Hollman et al., 2011; Kerimi and Williamson, 2016).

To test these above anomalies and associations further, we have undertaken a exploratory analyses of additional samples collected from a recently completed dietary intervention study which used 2 distinct doses mixed spices (Haldar et al., 2017). Spices are naturally rich in polyphenolic antioxidants (Shan et al., 2005; Yashin et al., 2017), although very few well-controlled randomized trials have investigated the effects of spice consumption on *in vivo* redox status in humans (Srinivasan, 2014). Furthermore, previous studies have suggested the utility of allantoin as a biomarker of oxidative stress (Grootveld and Halliwell, 1987; Benzie et al., 1999; Gruber et al., 2009) although, controlled dose response studies with dietary antioxidants to test this in humans have been limited. We have therefore investigated whether consumption of polyphenol rich curry, in two separate doses can modulate postprandial plasma concentrations of allantoin and allantoin to uric acid ratio and compared them with a more established marker, i.e., 8-*iso* Prostaglandin F_2α_ (F2-isoprostanes). Given the intricate link between postprandial glucose homeostasis, oxidative stress, and endothelial function (Ceriello et al., 2002; Brownlee, 2005), we also measured endothelial function using peripheral arterial tonometry (endoPAT).

## Methods

The details of this randomized controlled crossover trial have been previously described (Haldar et al., 2017). The study was registered at www.clinicaltrials.gov (Identifier No. NCT02599272) and was approved by the Domain Specific Research Board (DSRB) ethics committee, Singapore (Reference: C/2015/00729) and conducted according to the Declaration of Helsinki based good clinical practice guidelines. All volunteers provided written, informed consent to participate in this study. In brief, 17 healthy, non-smoking, Chinese men, aged 23.7 ± 2.44 years and BMI 23.1 ± 2.31 kg/m^2^ completed all 3 doses of the study. The volunteers were asked to consume one of three test meals for breakfast, after an overnight fast, consisting of either non-curry Dose 0 Control (D0C), or Dose 1 Curry (D1C) or Dose 2 Curry (D2C) treatments containing 0, 6, and 12 g mixed spices, respectively. The mixed spices preparations for D1C and D2C were identical and were prepared by thoroughly mixing dried powders of different spices consisting of turmeric, coriander seeds, cumin seeds (all Everest Spices, India), dried Indian gooseberry (“*amla,” emblica officinalis*, Ramdev Spices, India), cayenne pepper (Robertson’s, South Africa), cinnamon (McCormick’s, United States), and clove (Robertson’s, South Africa) and were mixed in the ratio of 8:4:4:4:2:1:1, respectively. Additionally, the test meals contained same total amounts of vegetables (180 g per portion), with D0C consisting of 130 g peeled eggplant and 50 g tomato puree, D1C made of 90 g peeled eggplant and 90 g “curry base vegetables” (made from tomato, onion, ginger and garlic in ratio of 5:2:1:1) and D2C made of 180 g curry base vegetables, in the same ratio as D1C. There were a minimum of 1 week washout period between each treatment. The meals were matched for total energy contents and macronutrient compositions. The total polyphenol contents (TPC) of the test meals were 130 ± 18.7 mg GAE (gallic acid equivalent), 556 ± 19.7 mg GAE and 1113 ± 211.6 mg GAE for D0C, D1C, and D2C, respectively, as measured using the Folin-Ciocalteu assay, as described previously (Haldar et al., 2017).

Blood samples for the measurements of the markers of oxidative stress were collected at 0 h (baseline), immediately prior to test meal consumption and then at 2 and 3 h following test meal consumption. Plasma samples were analyzed for 8-iso Prostaglandin F2α (F2-isoprostanes), allantoin and uric acid (UA) using a LCMS/MS (Agilent Technologies) method, developed in-house, as detailed in the Supplementary Table 1. All biological samples were analyzed in a blinded manner. Endothelial function was measured using the Endo-PAT 200 device (Itamar Medical, Israel) based on minor modifications of a previously published protocol (Moerland et al., 2012). In brief, the volunteers rested in a supine position in a quiet thermostatic room (maintained at 23°C), under minimal lighting, for 10 min following which 5 min of “baseline” arterial blood flow measurements in the index fingers of both arms were undertaken using a pair of probes fitted to each finger. After this, the arterial blood-flow was restricted for a period of 5 min (the “occlusion period”) using a sphygmomanometer cuff maintained at a pressure of 200 mm Hg in the “test arm,” whereas no intervention was undertaken in the other “control” arm. Immediately following this “occlusion period” in the test arm, the “post-occlusion period” arterial blood flow measurements were undertaken in both index fingers for the final 5 min. The reactive hyperemia index (RHI), which is a measure of the ratio of the arterial blood-flow between the test and control arms during the post-occlusion period relative to the baseline period was then automatically calculated using the device’s built-in software, as a measure of endothelial function. Two measurements were undertaken during each study session: one at 0 h and another at 2 h. The effects of various test meals on the change from baseline (0 h) values at 2 and 3 h (where applicable) were tested using two-way repeated measures ANOVA with treatment (dose) and time as the main effects and change from baseline as the dependent variable. The data were tested for normality using the Shapiro-Wilk test and Bonferroni corrections were applied for multiple treatments while undertaking pairwise comparisons. All statistical analysis in this study was done using Statistical Package for the Social Sciences (IBM SPSS version 24, IBM Corp, Armnok, NY, United States) and two-tailed statistical significance test was set at α = 0.05. Considering that this study was hypothesis generating, no *a priori* formal sample size calculation was undertaken for the outcome measures reported here.

## Results

**FIGURE 1:**
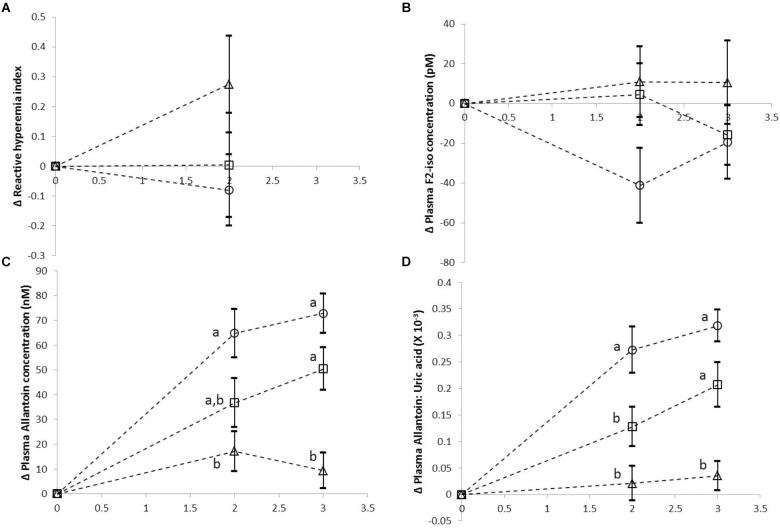
Mean change from baseline (±SEM) for reactive hyperaemia index **(A)**, plasma F2-isoprostane concentration **(B)**, plasma allantoin concentration **(C)**, and plasma allantoin to uric acid ratio **(D)** for each dose of curry. Open circles 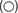 represents Dose 0 Control, open squares 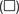 represents Dose 1 Curry and open triangles (Δ) represent Dose 2 curry. The data is presented here for 17 volunteers who completed all 3 doses of the study. Different letters indicate significant differences between various doses at any given time point.

The mean (±SEM) of change from baseline (0 h) values for various test meals (treatments) taken during various time points are shown in Figures 1A–D. Data from 17 participants who completed all 3 doses of the study are presented here. As shown in Figure 1, there was no significant difference between the 3 treatments in the post-meal change from baseline RHI, measured at 2 h. Similarly, there was no significant difference between treatments in the change from baseline plasma F2-isoprostanes concentrations during post meal (2 and 3 h) time points. However, we found significant lowering of the increase from baseline in the postprandial plasma allantoin concentration, as well as in plasma allantoin to uric acid ratio with increasing doses of curry intake (both *p* < 0.001).

## Discussion

Previous reports have suggested associations between postprandial oxidative stress and risk of several chronic diseases including type 2 diabetes and cardiovascular diseases (Sies et al., 2005). Our trial was one of the first dose-response studies with polyphenol rich mixed spices, in dietary doses, investigating their effects on two separate markers of postprandial redox balance *in viv*o as well as on endothelial function in healthy volunteers. While two separate, similar but independent, single-dose trials using the same mixed herbs and spices mix added to hamburgers previously found significant reductions in other markers of postprandial oxidative stress such as plasma malondialdehyde (MDA) concentration in healthy volunteers (Li et al., 2013) as well as improvements in endothelial function, and decreases in urinary MDA excretion in type 2 diabetics, we did not observe any effects using our curry preparations on either plasma F2-isoprostanes or on endothelial function. However, it should be noted that the mean intra-measurement variability for EndoPAT measurement at baseline across 3 doses was about 21%, which may have been a limitation for this measurement. However, we did observe significant dose-dependent reductions in the increase in postprandial allantoin concentration from baseline (as well as on the allantoin to uric acid ratio).

The findings of our study suggest that allantoin may be sensitive to acute changes in redox balance *in vivo*, as observed following the consumption of polyphenol rich curry at 2 distinct doses. Allantoin is produced in humans only via the non-enzymatic oxidation of uric acid (UA), since humans do not express the uricase enzyme (Kaur and Halliwell, 1990; Kand’ár et al., 2006). UA has long been recognized as a potent antioxidant *in vivo* (Ames et al., 1981), including its role to maintain levels of other antioxidants in the body such as ascorbic acid (Sevanian et al., 1991), as well as reacting with several oxidants *in vivo* (Becker, 1993). Furthermore UA have been shown to contribute a large proportion toward the total antioxidant capacity of plasma (Benzie and Strain, 1996) and increased oxidation products of UA, i.e., allantoin have also been found in several disease states associated with elevated oxidative stress (Grootveld and Halliwell, 1987; Özkan et al., 2006; Chung and Benzie, 2013), as well as following acute bouts of oxidative stress generating activities such as smoking (Seet et al., 2011) and exercise (Kandar et al., 2014), although fewer studies investigated changes in plasma allantoin concentrations following acute dietary modulations. On the contrary, while plasma F2-isoprostanes is a long established marker of oxidative stress, specifically of lipid peroxidation *in vivo*, the utility of this measurement in population monitoring studies is somewhat limited, partly due to the methodological rigor required in the sample preparation and analyses, which have given rise to inconsistent results between various studies (Milne et al., 2015). Moreover, there may have been other biological reasons for our study observations. This study was undertaken in young, healthy and non-smoking volunteers who probably already had an adequate antioxidant defense *in vivo*. Therefore, the body’s antioxidant defenses, including UA were utilized as the first line of defense in conditions of increased postprandial oxidative stress within normal physiological range, in order to maintain a healthy redox-balance. Thus, in such conditions, oxidation of macromolecules (including lipid peroxidation) were adequately prevented irrespective of the total antioxidant/polyphenol contents of the test meals. Hence, we did not observe any noticeable effects on postprandial F2-isoprostanes or on changes in vascular function with or without curry intake. While some of the polyphenols in spices, including the well-studied curcumin is poorly bioavailable, although we have recently shown that some of the secondary metabolites of polyphenols, such as cinnamic acid, with high antioxidant activity *in vitro*, did increase in plasma with increasing doses of curry intake (Haldar et al., 2018). Furthermore, given the large inter- and intra-individual variability in the responses in these measurements, there is a possibility that our study was not adequately powered to detect subtle differences as shown in detail in Supplementary Table 2, which included the effect sizes and the raw mean differences, along with the *p*-values to enable the readers to make an informed judgment. Nonetheless, the dose dependent decreases in postprandial allantoin with increasing curry doses does indicate that the phenolic compounds in our curry mix prevented oxidation of certain endogenous antioxidants, such as UA, thereby maintaining an improved antioxidant status *in vivo*. Thus, allantoin may have a utility as a biomarker of redox balance, in an acute setting although further studies with larger population size and/or in longer term settings and with more comprehensive comparisons with other classic redox balance markers are warranted.

## Data Availability Statement

The raw data supporting the conclusion of this manuscript will be made available by the authors, without undue reservation, to any qualified researcher.

## Author Contributions

SH, CH, and CD designed the study and produced the hypothesis. LP performed the LCMS/MS sample analyses. SC and JL undertook the dietary intervention. SP performed the statistical analysis of the data. All authors wrote and approved the manuscript.

## Conflict of Interest Statement

The authors declare that the research was conducted in the absence of any commercial or financial relationships that could be construed as a potential conflict of interest. The handling Editor declared a shared affiliation, though no other collaboration, with several of the authors SH, JL, SC, SP, and CH at the time of review.
