# Supplementary material for: Reductions in Postprandial Plasma Allantoin Concentrations With Increasing Doses of Polyphenol Rich Curry Intake – A Randomized Crossover Trial
Source: Front Physiol. 2019 Jan 9;9:1899. doi: 10.3389/fphys.2018.01899 (PMC6333854; doi:10.3389/fphys.2018.01899)
Supplement: Supplementary file 1 [file Table_1.DOCX]

Supplementary Material

**Reductions in postprandial plasma allantoin concentrations with increasing doses of polyphenol rich curry intake – a randomized crossover trial**

**Sumanto Haldar^*^, Leroy Sivappiragasam Pakkiri, Joseph Lim, Siok Ching Chia, Shalini Ponnalagu, Chester Lee Drum, Christiani Jeyakumar Henry**

*** Correspondence:** Corresponding Author: [sumanto_haldar@sics.a-star.edu.sg](mailto:sumanto_haldar@sics.a-star.edu.sg)

# Supplementary Tables

**SUPPLEMENTARY TABLE 1: LCMS/MS methods for the determination of plasma concentrations of F2-isoprostanes allantoin and uric acid**

| **Sample Preparation for LCMS/MS**  Human plasma samples (50 μL) were aliquoted into a 96‐well plate, then spiked with 50 μL of 1 μg/mL of Allantoin (AL) and Uric acid (UA), and 0.5 ng/mL of 8-*iso* Prostaglandin F_2α_ (F2-isop) heavy isotope standards. After treating with 360 μL of ice-cold Acetonitrile containing 0.1% Formic acid, the plate was mixed on a shaker at 1000 rpm/min for 10 minutes, then centrifuged down at 2270*g* for 50 minutes at 4°C. 140 μL of the supernatant was carefully transferred to a 96‐microwell plate and loaded into the auto‐sampler for analysis by LCMS/MS. Ion counts were then normalized against that of the heavy isotope standard, before using the standard curve for quantification.  **LCMS/MS Method for** **Allantoin, Uric acid and** **8-*iso* Prostaglandin F_2α_**  We developed and validated LCMS/MS methods for All, UA and F2-isop assays. Liquid chromatographic (LC) separation of these biomarkers was carried out on an Agilent 1290 Infinity II LC system (Agilent Technologies, Santa Clara, CA) with PEEK coated SeQuant^®^ZIC^®^-cHILIC 3mm, 100Å 100 x 2.1 mm HPLC column (Merck Pte Ltd, Singapore) maintained at 40°C.  The organic solvent was Acetonitrile containing 0.1% Formic acid (Solvent A) and the aqueous solvent used was 20 mM Ammonium Formate pH 4.0 (Solvent B). A linear LC gradient on Binary Pump (Agilent Model G7120A) was set up with percentage of Solvent B as follows: 10% at 0 minute, 70% at 9.00 minutes, 70% at 11.0 minutes, and 10% between 11.1 and 11.5 minutes, with flow rate of 0.4 mL/min. The column was further equilibrated for further 11.5 minutes with 10% Solvent B. An additional high speed pump, Binary Pump B (Agilent Model G7120A), together with a Quick-Change valve head, 2-position/10-port, 1,300 bar (Agilent Part No: 5067-4240), were utilized to reduce the cycle times by automated alternating column regeneration. % of Solvent B on Binary Pump B was maintained at 10% with flow rate of 0.4 mL/min. The sample injection volume was 5 μL.  For mass detection, the LC eluent is connected online to an Agilent 6495 Triple Quadrupole MS system (Instrument model G6495A, Agilent Technologies) operated with the electrospray source in negative ionization mode. The electrospray ionization source conditions were as follows: capillary voltage of 4.0 kV, nozzle voltage of 500 V, iFunnel parameter high/low pressure RF of 90 V, nebulizer pressure of 60 psi, gas temperature of 290°C, sheath gas temperature of 350°C, Nebulizer was 35 psi, and sheath gas flow of 12 L/min.  Allantoin (Cat. # 05670), Uric Acid (Cat. # U2625), Uric acid-1,3-^15^N_2_ (Cat. # 4907997) were purchased from Sigma-Aldrich (Singapore), Allantoin-13C2,15N4, (Cat. # A540502), was purchased from Toronto Research Chemicals (Ontario, Canada), 8-*iso* Prostaglandin F_2α_ (Item №: 16350), and 8-iso Prostaglandin F2α-d4 Item №: 316350 were purchased from **I-DNA Biotechnology PTE LTD. These reagents were** used as standards for development of multiple reaction monitoring methods for quantification.  The multiple reaction monitoring (MRM) used for All and its heavy isotope standard were 157→114 with collision energy of 8 eV and Collision Accelerator Voltage (CAV) of 3V and 162.9→117.8 with collision energy of 20 eV and CAV of 5V, respectively. The MRM used for UA and its heavy isotope standard were 167→123.9 and 169→125.1, with collision energy of 13 eV and CAV of 3V, respectively. The MRM used for F2-isop and its heavy isotope standard were 353.4→193.2 and 357.4→197.1, with collision energy of 30 eV and CAV of 35, respectively.  For the quantification of All, a calibration curve was constructed by serial dilution using Optima^®^ LC/MS water (Fisher Chemicals) at concentrations of 9885, 4946, 1233, 620, 154, 79.7, 38.6, 9.49, 4.74, 2.37 nM. The All QCs concentrations were 2473, 306, 19.6 nM. A linear curve with a weightage of 1/X was plotted, and the R-Squared value was 0.99541094.  Similarly, calibration curves for UA and F2-isop were constructed. The UA standards were 744, 372, 186, 93, 46.5, 23.5, 11.6, 5.81, 2.91 uM. The R2 value for the linear curve was 0.98967018. The F2-isop standards were 2697, 1917, 358, 202, 67.1, 11.1 pM, and the QCs were 55.8 and 12.3 pM. The R2 value for the linear curve was 0.99114154. |
| --- |

**SUPPLEMENTARY TABLE 2: Summary statistics for each measurement. The p-values were obtained from two-way repeated measures ANOVA with time and doses as the main effects and change from baseline as the dependent variable. The p-values for the pairwise comparison (within each measurement) have been obtained using Bonferroni correction.**

| Measurement | Statistical test | Mean difference | Effect size | Significant? | P-value |
| --- | --- | --- | --- | --- | --- |
| Allantoin: Uric Acid concentration | Main effect of time |  | 0.201^a^ | No | 0.062 |
|  | Main effect of doses |  | 0.599 | Yes | <0.001 |
|  | Main effect of interaction |  | 0.039 | No | 0.527 |
|  | Dose 0-Dose 1 | 0.128 (SE^c^ = 0.038), CI^d^ : (0.027, 0.229) | 0.817^b^ | Yes | 0.011 |
|  | Dose 0 -Dose 2 | 0.267 (SE=0.041), CI: (0.158, 0.377) | 1.579 | Yes | <0.001 |
|  | Dose 1-Dose 2 | 0.139 (SE=0.037), CI: (0.040, 0.239); | 0.911 | Yes | 0.005 |
| Allantoin concentration | Main effect of time |  | 0.045 | No | 0.398 |
|  | Main effect of doses |  | 0.504 | Yes | <0.001 |
|  | Main effect of interaction |  | 0.097 | No | 0.195 |
|  | Dose 0-Dose 1 | 25.231 (SE=9.109), CI: (0.882, 49.581) | 0.672 | Yes | 0.041 |
|  | Dose 0 -Dose 2 | 55.511 (SE=10.687), CI: (26.943, 84.078) | 1.260 | Yes | <0.001 |
|  | Dose 1-Dose 2 | 30.279 (SE=9.362), CI:(5.255, 55.304) | 0.784 | Yes | 0.016 |
| F2-isoprostane concentration | Main effect of time |  | 0 | No | 0.979 |
|  | Main effect of doses |  | 0.113 | No | 0.147 |
|  | Main effect of interaction |  | 0.066 | No | 0.338 |
|  | Dose 0-Dose 1 | -24.883 (SE=21.229), CI: (-81.629, 31.863) | 0.284 | No | 0.775 |
|  | Dose 0 -Dose 2 | -41.449 (SE=21.531), CI: (-99.0, 16.10) | 0.467 | No | 0.217 |
|  | Dose 1-Dose 2 | -16.566 (SE=19.154), CI: (-67.765, 34.634) | 0.210 | No | 1 |
| Reactive Hyperemia Index (RHI) | Main effect of time | nil |  |  |  |
|  | Main effect of doses |  | 0.04 | No | 0.523 |
|  | Main effect of interaction | nil |  |  |  |
|  | Dose 0-Dose 1 | 0.003 (SE=0.219), CI: (-0.583, 0.589) | 0.003 | No | 1 |
|  | Dose 0 -Dose 2 | -0.228 (SE=0.193), CI: (-0.743, 0.287) | 0.286 | No | 0.764 |
|  | Dose 1-Dose 2 | -0.231 (SE=0.271), CI: (-0.956, 0.495) | 0.206 | No | 1 |

^a^ Effect sizes of the main effects are Partial eta squared values as returned by SPSS.

^b^ Effect size for the mean differences in change scores between the doses are calculated using method of Cohen’s d_z_ (t / $\sqrt{n}$ ), n in this case is equal to 17.

^c^ SE: Standard error of the mean differences in change scores between the doses.

^d^ CI: 95% confidence interval of the mean differences in change scores between the doses.
